# Insomnia in Chinese College Students With Internet Addiction: Prevalence and Associated Clinical Correlates

**DOI:** 10.3389/fpsyt.2020.596683

**Published:** 2020-11-27

**Authors:** Yanmei Shen, Xingyue Jin, Yaru Zhang, Chunxiang Huang, Jianping Lu, Xuerong Luo, Xiang Yang Zhang

**Affiliations:** ^1^Department of Psychiatry, The Second Xiangya Hospital, Central South University, Changsha, China; ^2^Hunan Key Laboratory of Psychiatry and Mental Health, China National Clinical Research Center on Mental Disorders (Xiangya), China National Technology Institute on Mental Disorders, Hunan Technology Institute of Psychiatry, Mental Health Institute of Central South University, Changsha, China; ^3^Department of Child and Adolescent Psychiatry of Shenzhen Kangning Hospital, Shenzhen Mental Health Center, Shenzhen, China; ^4^Chinese Academy of Sciences (CAS) Key Laboratory of Mental Health, Institute of Psychology, Beijing, China

**Keywords:** Chinese college students, risk factors, prevalence, internet addiction, IA, insomnia

## Abstract

**Background:** Internet addiction (IA) has gained more and more attention for its negative impact on the subjects' study and daily life. However, in a large sample, there is little research on the association between IA and insomnia in Chinese college students. This study aimed to investigate the prevalence of insomnia and its related risk factors among Chinese college students with IA.

**Methods:** A cross-sectional design was used to investigate 627 Chinese college students with IA. Each student completed a survey on demographic data, Internet addiction (Revised Chinese Internet Addiction Scale), depression (Self-Rating Depression Scale), insomnia (Athens Insomnia Scale), anxiety (Self-Rating Anxiety Scale), and suicidal behavior. Binary logistic regression analysis was employed to adjust for confounding factors.

**Results:** The prevalence of insomnia among students with IA was 54.86%. Compared with IA students without insomnia, IA students with insomnia were more likely to be younger, smoking, drinking, have anxiety, depression, suicidal ideations, suicide plans, and suicide attempts (all *p* < 0.05). Moreover, drinking [OR, 1.664; 95% confidence interval (CI), 1.139–2.431; *p* = 0.008], anxiety (OR, 2.321; 95% CI, 1.116–4.826; *p* = 0.024), and suicidal ideation (OR, 1.942; 95% CI:1.295–2.911; *p* = 0.001) were independently associated with insomnia in IA students.

**Conclusions:** Insomnia is very common in Chinese IA students. Drinking, anxiety, and suicidal ideation are independently correlated with insomnia. This study provides valuable evidence for school counselors and clinical professionals to assess Internet addiction, insomnia, and suicide risk.

## Introduction

Internet addiction (IA) refers to the excessive use of the Internet, which leads to damage to personal psychological states (1). Participants with IA are often unable to control their desire to surf the Internet, escape from a dysphoric mood, and lose interest in other things (2, 3). With the development of the Internet and multimedia, the use of the Internet is becoming an essential part of people's daily life. It is reported that by 2010, the amount of global Internet users has reached 4.39 billion (4). Internet addiction has attracted more and more attention. A meta-analysis conducted in 31 countries and seven world regions showed that the pooled prevalence of IA was 6.0% (5). The Internet has brought enormous convenience to people's life. It is estimated that Chinese Internet users had reached 854 million by June 2019 (6). Millions of people use the Internet to send messages (825 million), browse news (686 million), make online purchase (639 million), order take-outs (421 million), or watch videos (759 million) (6). Despite the benefits of the Internet, spending too much time on the Internet can also have a negative impact on subjects' study, work, and daily life. Studies have shown that people with IA usually have poorer academic performance (7), lower happiness (8), and are more likely to feel lonely (9). In addition, adolescents with IA are characterized by anger, distress, social withdrawal, familial conflict, and loss of control, and they are more likely to have personality disorders (10). Studies have also indicated that IA is associated with mental health problems, such as insomnia (11, 12), anxiety (13), depression (13), bipolar disorders (14), self-harm, and suicidal behavior (13). IA has become the main concern of the world society today.

Insomnia refers to the difficulty of initiating and/or maintaining sleep, or early morning awakening, which can cause damage to the individual's daily life, such as fatigue or daytime sleepiness (15). Previous studies have revealed the link between IA and insomnia. For example, Li et al. observed a higher prevalence of insomnia among middle school students with IA (16). A meta-analysis indicated that compared with those subjects without IA, subjects with IA were 2.2 times more likely to suffer from insomnia [overall pooled odds ratio (OR) = 2.20] and present with shorter sleep duration [overall pooled standardized mean difference (SMD) = −0.24 h] (17). Further, studies have shown that individuals with IA show a higher proportion of sleep disorders, such as shorter sleep duration (18, 19), longer sleep duration required (18), poor sleep quality (18–20), excessive daytime sleepiness (21), and sleep–wake behavior problems (21).

Compared with younger students, college students have more free time after studying. In addition, courses and homework in universities often need to be finished with the help of the Internet. Therefore, college students have more opportunities to surf the Internet and are susceptible to Internet addiction. Sleep disorders such as insomnia for college students with IA are also issues of great concern to us. Previous studies have reported that college students with IA have significantly reduced sleep quality and quantity (22, 23). On the other hand, Kitazawa et al. reported that Japanese college students with poor sleep quality were 1.52 times more likely to have IA (22). Generally speaking, although previous studies have revealed the relationship between IA and insomnia, most of the studies have mainly focused on young people, particularly the teenagers in foreign countries; however, there is little research on Chinese college students. As far as we know, our study was the first of its kind to investigate the relationship between IA and insomnia in a large sample of Chinese college students. The main purpose of this study was to investigate the prevalence of insomnia and its related risk factors among IA college students in China.

## Methods

### Participants

A cross-sectional design was adopted in this study. The Chinese Internet addiction scale (CIAS-R) was utilized to screen for students with IA with convenience sampling method. The CIAS-R was delivered online anonymously to students from the Technology and Changsha Medical University and Changsha University of Science. Students completed the scale and present with a CIAS-R total score reached, or more than 53 were enrolled in this study. Therefore, this study enrolled 627 college students with IA. The inclusion criteria for participation were 1) participants were between 17–25 years old; 2) their score on the Chinese Internet addiction scale (CIAS-R) reached or exceeded 53; 3) they were in good health; and 4) they agreed to sign informed consent to participate in this study. All participants were informed of their right to decide to take part in or quit. They were also informed of their right to refuse to participate, and they can withdraw from this study at any time. The survey was provided online through social media app (WeChat). In addition, the survey was delivered anonymously to protect the privacy of the students. College counselors were responsible to guide the students to fill the survey online, and they were trained on how to collect the data before providing guidance. This study was approved by the Ethics Committee of the Second Xiangya Hospital. Each college students provided informed consent before taking part in this study.

### Assessment

Participants' sociodemographic data, including sex, age, community, nationality, one-child family, parents' education level, good relationship with mother (yes or no), good relationship with father (yes or no), family income, smoking, drinking habits, right handedness, history of mental disorders, history of physical disorders, family history of mental disorders (FHMD), and suicidal behaviors (suicidal ideation, suicide plans, and attempts) were collected by trained college counselors.

Internet addiction was assessed by the revised Chinese Internet addiction scale (CIAS-R); CIAS-R is a 19-item self-report questionnaire designed to measure participants' addiction to the Internet. Each item of the scale was rated from 1 (1 for complete inconformity) to 4 (complete conformity). The total score of CIAS-R ranges from 0 to 76. When the total score of CIAS-R reached or exceeded 53, the students were divided into Internet addiction group. According to reports, CIAS-R has good psychometric properties (24). When their score in the CIAS-R reached or exceeded 53, they were regarded as having IA.

Participants' anxiety and depression were measured by the Self-rating Depression Scale (SDS) and Self-rating Anxiety Scale (SAS) (25, 26). The cutoff score for anxiety was 50 on SAS, and the cutoff score for depression was 53 on SDS (27). SAS and SDS were widely used and show good psychometric properties among the Chinese population (28, 29).

The Athens Insomnia Scale (AIS) was utilized to measure participants' insomnia symptoms. AIS is an eight-item self-report scale, and each item has a four-point Likert scale from 0 to 3 (0 for no problem or equivalent, and 3 for very severe problem or equivalent). The total score of AIS is between 0 and 24, the cutoff score of insomnia is 6 (30). A higher AIS score indicates a higher level of insomnia symptoms. It is reported that AIS has good psychometric properties in the Chinese population (30).

### Statistical Analyses

In this study, chi-squared test was employed to compare the group differences for categorical variables, and t-test was utilized for continuous data (continuous data conformed to a normal distribution). The odds ratio (OR) was calculated through the univariate logistic analysis. Binary logistic regression analysis was employed to adjust for confounding factors (adjusted OR); all of the variables in Table 1 were put into the binary logistic model. The significance level of this study was set at two-tailed p value of 0.05. IBM SPSS 22 was utilized to perform all the statistical analyses.

**Table 1:** Demographics and clinical characteristics between participants with and without Internet addiction (IA) and between IA students with and without insomnia.

**Variables**	**IA with insomnia (*n* = 344)**	**IA without insomnia (*n* = 283)**	***p***	
Age (years), mean (SD)	20.12 (1.525)	20.25 (1.50)	0.047^*^	
**Sex**				
Males, *n* (%)	144 (41.9%)	115 (40.6%)	0.757	
Females, *n* (%)	200 (58.1%)	168 (59.4%)		
**Community urban**				
Urban	135 (39.2%)	119 (42.0%)	0.476	
Rural	209 (60.8%)	164 (58.0%)		
**Nationality**				
Han	305 (88.7%)	249 (88.0%)	0.793	
Others	39 (11.3%)	34 (12.0%)		
One-child family	122 (35.5%)	109 (38.5%)	0.431	
Good relationship with mother	323 (93.9%)	269 (95.1%)	0.530	
Good relationship with father	301 (97.5%)	261 (92.2%)	0.053	
Smoking	54 (15.7%)	18 (6.4%)	0.000^***^	2.741 (1.568–4.794)
Alcohol	158 (45.9%)	92 (32.5%)	0.001^**^	1.764 (1.272–2.445)
**Family income/year (yuan)**				
Less than 30,000	81 (23.5%)	74 (26.1%)	0.612	
30,000~70,000	160 (46.5%)	121 (42.8%)		
More than 70,000	103 (29.9%)	88 (31.1%)		
**Father's education level**				
JMSB	172 (50.0%)	143 (50.5%)	0.976	
HSTS	125 (36.3%)	103 (36.4%)		
CUA	47 (13.7%)	37 (13.1%)		
**Mother's education level**				
JMSB	204 (59.3%)	170 (60.1%)	0.938	
HSTS	110 (32.0%)	87 (30.7%)		
CUA	30 (8.7%)	26 (9.2%)		
Right-handedness	300 (87.2%)	252 (89.0%)	0.481	
Physical disorder history	24 (7.0%)	14 (4.9%)	0.289	
Mental disorder history	13 (3.8%)	4 (1.4%)	0.070	
FHMD	14 (4.1%)	5 (1.8%)	0.094	
Anxiety	197 (57.3%)	77 (27.2%)	0.000^***^	3.585 (2.557–5.027)
Depression	172 (50.0%)	63 (22.3%)	0.000^***^	3.492 (2.459–4.960)
Suicidal ideation	192 (55.8%)	102 (36.0%)	0.000^***^	2.241 (1.623–3.095)
Suicide plans	52 (15.1%)	22 (7.8%)	0.005^**^	2.113 (1.249–3.574)
Suicide attempts	96 (27.9%)	38 (13.4%)	0.000^***^	2.496 (1.648–3.780)

*IA, Internet addiction; JMSB, junior middle school and below; HSTS, high school or technical school; CUA, college or university and above; FHMD, family history of mental disorder*.

* p < 0.05;

** p < 0.01;

*** *p < 0.001*.

## Results

Table 1 shows that the prevalence of insomnia among students with IA was 54.86% (344/627). IA students with insomnia were younger, and more likely to smoke, drink alcohol, suffer from depression or anxiety, used to have suicidal thoughts, make suicide plans, and with a history of committing suicide (all p < 0.05). In addition, no significant group difference was observed in sex, nationality, one-child family, good relationship with parents, family income, parents' education level, right-handedness, physical disorder history, mental disorder history, and family history of mental disorder (FHMD). The following variables remained to be significant after controlling for covariate variables in binary logistic analysis: drinking [OR, 1.664; 95% confidence interval (CI), 1.139–2.431; p = 0.008], anxiety (OR, 2.321; 95% CI, 1.116–4.826; p = 0.024), and suicidal ideation (OR, 1.942; 95% CI: 1.295–2.911; p = 0.001) (see Table 2).

**Table 2:** Multivariate analysis for variables associated with Internet addiction participants with insomnia.

**Variable**	**B**	**S.E**.	**Wald**	**df**	**Sig**.	**Exp(B)**	**95%CI for EXP(B)**	
							**Lower**	**Upper**
Age	0.061	0.059	1.075	1	0.300	1.063	0.947	1.193
Sex	0.107	0.194	0.304	1	0.582	1.113	0.761	1.626
Community	0.199	0.227	0.771	1	0.380	1.221	0.782	1.905
Nationality	−0.251	0.274	0.837	1	0.360	0.778	0.455	1.332
One-child family	−0.048	0.209	0.053	1	0.818	0.953	0.633	1.435
Good relationship with mother	0.827	0.450	3.377	1	0.066	2.286	0.947	5.519
Good relationship with father	−0.488	0.337	2.097	1	0.148	0.614	0.317	1.188
Smoking	0.601	0.330	3.315	1	0.069	1.825	0.955	3.486
Alcohol	0.509	0.193	6.934	1	0.008^**^	1.664	1.139	2.431
Family income			1.679	2	0.432			
Family income 1	0.290	0.224	1.678	1	0.195	1.336	0.862	2.073
Family income 2	0.193	0.260	0.550	1	0.458	1.213	0.728	2.019
Mother's education level			1.070	2	0.586			
Mother's education level 1	−0.251	0.243	1.069	1	0.301	0.778	0.483	1.252
Mother's education level 2	−0.216	0.449	0.231	1	0.631	0.806	0.334	1.942
Father's education level			1.212	2	0.546			
Father's education level 1	0.246	0.225	1.196	1	0.274	1.279	0.823	1.989
Father's education level 2	0.245	0.399	0.377	1	0.539	1.278	0.584	2.793
Right-handedness	0.122	0.288	0.181	1	0.671	1.130	0.643	1.986
Physical disorder history	−0.343	0.435	0.621	1	0.431	0.710	0.303	1.665
Mental disorder history	0.553	0.732	0.571	1	0.450	1.739	0.414	7.298
FHMD	0.877	0.644	1.855	1	0.173	2.404	0.680	8.490
Anxiety	0.842	0.374	5.081	1	0.024^*^	2.321	1.116	4.826
Depression	0.391	0.390	1.006	1	0.316	1.479	0.689	3.175
Suicidal ideation	0.664	0.207	10.317	1	0.001^**^	1.942	1.295	2.911
Suicide plans	−0.273	0.354	0.594	1	0.441	0.761	0.380	1.524
Suicide attempts	0.180	0.289	0.390	1	0.532	1.198	0.680	2.109

*CI, confidence interval; FHMD, family history of mental disorder; family income 1, 30,000–70,000; family income 2, more than 70,000; mother's education level 1, high school or technical school; mother's education level 2, college or university and above; father's education level 1, high school or technical school; father's education level 2, college or university and above*.

* p < 0.05;

** *p < 0.01*.

## Discussion

To the best of our knowledge, this study was the first of its kind to investigate the association between insomnia and IA in Chinese college students with a large sample size. We found that insomnia was very common among Chinese college students with IA, with a prevalence rate of 54.86%. We also revealed that insomnia was associated with younger age, smoking, drinking alcohol, anxiety, depression, suicidal thoughts, suicide plans, and suicide attempts. Moreover, we found that after controlling for confounding variables, drinking alcohol, anxiety, and suicidal ideation were still independent risk factors for insomnia in IA college students.

This study revealed that IA and insomnia coexisted at a high level (54.86%), which was similar to the prevalence rate reported in Hong Kong (52.7%) (19), but lower than that in Taiwan (63.4%) (12). This difference may be due to different measurement methods utilized in different studies. Another possible reason may be that each study recruited a different population. Their study recruited participants of all ages, while the subjects in our study were college students. In addition, previous studies conducted among college students have also revealed a significant association between IA and insomnia (11, 23, 31). There are several reasons that may have contributed to this association. Participants with IA are frequently reported to have poor time management skills, and excessive use of the Internet may lead to reduced sleep time (32). Furthermore, studies have shown that electronic devices such as computers and smartphones, can emit light, especially blue light, thereby suppressing individual's melatonin levels (33). Melatonin is secreted by the pinealocytes in the pineal gland and plays a very important role in human sleep (34).

We demonstrated that anxiety was independently associated with insomnia in IA students. The association between IA and anxiety was consistent with previous studies (11, 12, 22, 35, 36). For example, Evren et al. found that Turkish college students with a high probability of insomnia had higher levels of IA and anxiety (35). Younes et al. and Kitazawa et al. found that college students in Lebanon and Japan with potential IA or problematic Internet use had significantly higher anxiety levels than normal Internet users. Moreover, having anxiety tendencies contributed to increased IA risk (11, 22). A meta-analysis demonstrated that IA was positively associated with anxiety, especially among young adults (19–39 years old). Moreover, people with anxiety were 2.7 times more likely to suffer from IA than those without anxiety (pooled OR = 2.70) (36). As for the relationship between anxiety and insomnia, Taylor et al. proposed that people with insomnia were 17.35 times more likely to suffer from anxiety than those without insomnia (37). The possible causes for the associations between insomnia and anxiety may include psychological factors, neural correlates of the brain, and genetic factors.

Psychologically, patients with insomnia usually exhibit higher levels of “neuroticism,” “internalization,” anxiety, and perfectionism-related traits (38), which may contribute to the susceptibility and permanence to insomnia. Insomnia patients often “keep their emotions to themselves,” which may lead to increased physiological activation (39) “Perfectionism” tends to set excessively high performance standards, which may lead to failure to fall asleep. Meanwhile, patients with insomnia are often characterized by excessive attention to sleep (40), decreased ability to concentrate (41), and daytime irritability (41), which may increase the likelihood of anxiety. In addition, treatment for insomnia can improve anxiety symptoms and vice versa (42). As for the neural substrates, previous studies have shown that the anterior cingulate cortex (ACC) is part of the pre-frontal network responsible for controlling amygdala activity from top to bottom (43). In patients with primary insomnia, the average connectivity between the left amygdala and right anterior cingulate cortex (rACC) decreases with increasing state anxiety, which means that decreased top–down control of the amygdala may increase the risk of developing anxiety disorder accompanied by preexisting primary insomnia (44). Moreover, the lower overall functional connectivity density values of the left anterior cingulate cortex (lACC) and right insula are associated with their anxiety level (45), but another study shows that the enhanced connectivity between the left insular lobe and the right anterior cingulate cortex (rACC) was negatively correlated with anxiety level (46). Therefore, more research is needed to explore the potential neural basis of the association between insomnia and anxiety. As for heredity, a twin study found that the genetic factors related to the etiology of insomnia overlap with those related to anxiety (42), suggesting that there is a biological genetic basis underlying insomnia and anxiety.

We also found that suicidal ideation was independently associated with insomnia in IA college students, which was consistent with previous studies (12, 20). For example, previous studies have found that poor sleep quality was associated with lifelong suicidal ideation (12) and lifelong suicide attempts (20) among people with IA. Many studies have identified insomnia as an independent, modifiable risk factor for suicide (47, 48). Studies have shown that treatments for insomnia, such as cognitive behavioral therapy for insomnia (CBT-I) (49) and controlled-release zolpidem (50) (a hypnotic drug) can reduce the risk of suicide (51). There are several reasons that may contribute to this association. Perlis et al. proposed a hypothesis that insomnia usually results in “hypofrontality” and diminished executive function, and then executive dysfunction can lead to difficulties in regulating moods, thoughts, or actions tend to think about things in a negative way and fail to inhibit inappropriate actions, such as suicidal ideation and suicide attempts (52). Similarly, McCall et al. found that false beliefs and attitudes toward sleep may be a specific mediator, making insomnia as a risk factor for suicidal ideation (53). Patients with insomnia are often mentally overactive and do not know how to handle it, and may have false ideas such as committing suicide to escape the disease.

This study should be interpreted with caution due to the following limitations. First, our research was a cross-sectional study, so we cannot draw any conclusions about causality. Future studies should employ a longitudinal research design to better explore the casual relationship between these variables. Second, despite the advantage of a large sample size, the participants in this study were from two universities in Hunan province, and more students from different places should be recruited in further studies to increase the representativeness. Third, this study employed self-report scales to measure the demographic and clinical features of participants, which may be biased compared to studies conducted utilizing diagnostic interview tools.

In conclusion, we have demonstrated that insomnia is very common among Chinese college students with IA. Further, this study revealed the association between insomnia and drinking alcohol, anxiety, and suicide ideation. These findings underscore the importance of addressing insomnia symptoms and related factors such as anxiety and suicide ideation among IA college students. Overall, this study provides valuable evidence for us to deepen our understanding of the pathological mechanisms underlying insomnia in the subjects with IA, and also provides a reference for school counselors and clinical professionals when assessing IA, insomnia and the risk of attempted suicide.

## Data Availability Statement

The data that support the findings of this study are available on request from the corresponding author. The data are not publicly available due to privacy or ethical restrictions.

## Ethics Statement

The studies involving human participants were reviewed and approved by the Ethics Committee of the Second Xiangya Hospital. The patients/participants provided their written informed consent to participate in this study.

## Author Contributions

XL, XZ, and YS were responsible for the study design. YS, XJ, and YZ were responsible for recruiting the participants. CH and JL were involved in statistical analysis. YS, YZ, and XJ were involved in manuscript preparation and drafting the paper. CH and JL were involved in editing and revising the manuscript. XL and XZ were responsible for the critical revision of the manuscript. All authors have contributed to and have approved the final manuscript.

## Conflict of Interest

The authors declare that the research was conducted in the absence of any commercial or financial relationships that could be construed as a potential conflict of interest.
